# Swallowing difficulties in adolescents: A case report and suggestion of treatment model

**DOI:** 10.1002/ccr3.6097

**Published:** 2022-08-11

**Authors:** Margareta Gonzalez Lindh, Emma Selander, Antti Juhani Kukka

**Affiliations:** ^1^ Department of Neuroscience Uppsala University Uppsala Sweden; ^2^ Centre for Research & Development Gävle Sweden; ^3^ Department of Pediatrics Region Gävleborg Sweden; ^4^ Uppsala Global Health Research on Implementation and Sustainability, Department of Women's and Children's Health Uppsala University Uppsala Sweden

**Keywords:** adolescent, avoidant restrictive food intake disorder, cognitive behavioral therapy, deglutition disorders, dysphagia, pediatrics

## Abstract

Dysphagia or difficulty swallowing in childhood necessitates multi‐disciplinary evaluation and management. This case report highlights the teamwork required for diagnostic work‐up to distinguish functional dysphagia from organic and psychiatric conditions in an adolescent girl. Treatment model based on cognitive behavioral therapy is also presented.

## Graphical abstract

Dysphagia in adolescents requires multi‐disciplinary approach for diagnosis and management. Sequential Oral Sensory feeding based on cognitive behavioral therapy is an option for managing behavioral dysphagia.

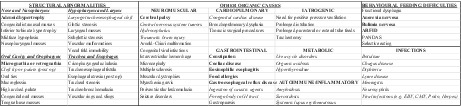

## 1 INTRODUCTION

**FIGURE 1:**
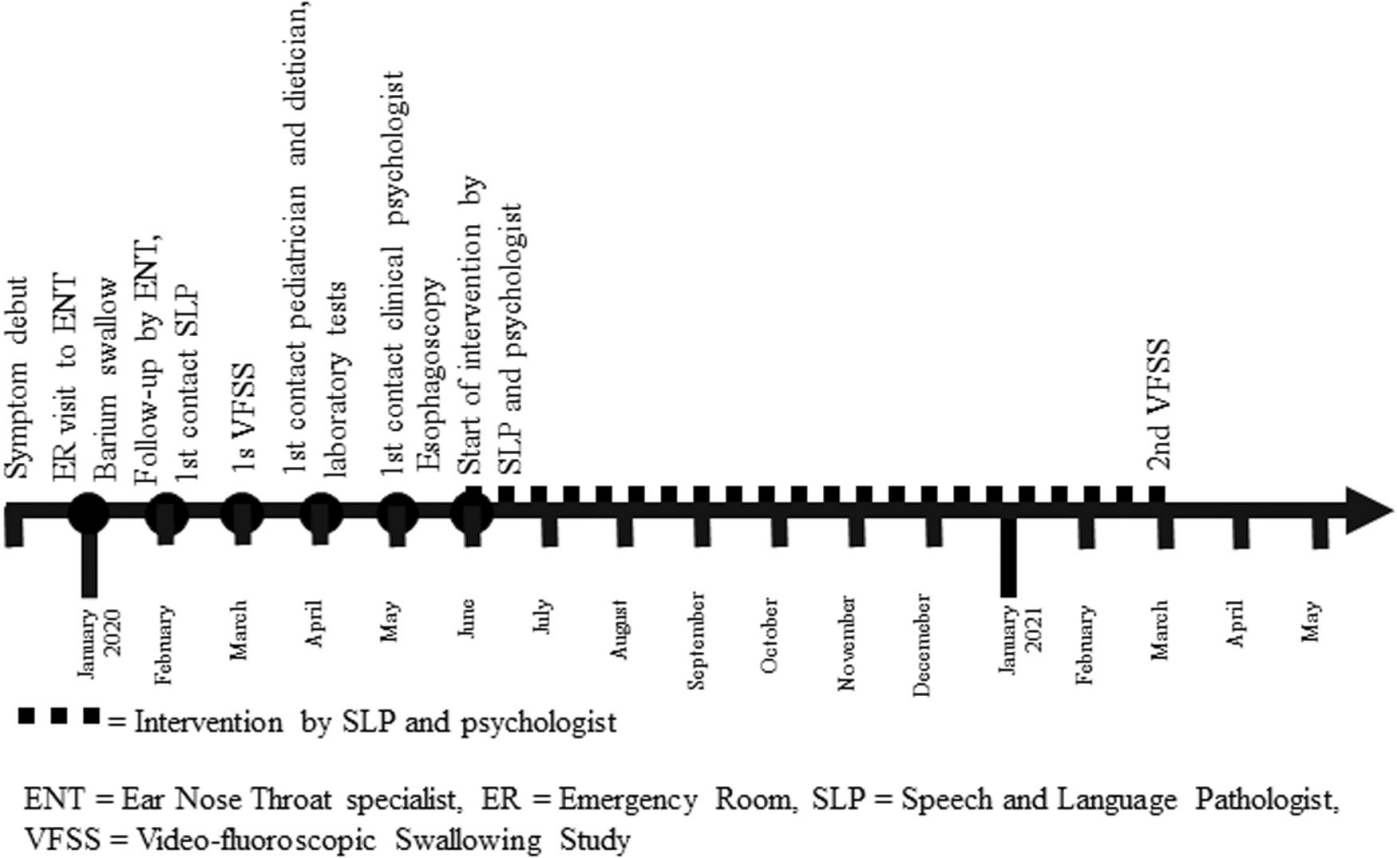
Case report time‐line

Dysphagia or difficulty swallowing is a relatively common problem in early childhood but decreases with increasing age.^1^, ^2^ Its prevalence depends on age group and underlying medical conditions. Up to a third of all infants have some form of feeding problem,^1^ whereas circa 1% of children aged 3–17 in the United States had some form of self‐reported swallowing problem.^2^ Dysphagia is considered one of the most common problems in children with cerebral palsy, neurological diseases and many syndromes.^3^ Distinguishing organic dysphagia from functional swallowing difficulties and other types of behavioral feeding problems is important for correct management, though these conditions often co‐exist.^4^ Although feeding, eating and swallowing are crucial for survival and both organic and functional dysphagia can severely impact nutritional intake, many hospitals have no clear guidelines on how to evaluate and manage this patient group and management is often done in an unsystematic way. Here we present a case highlighting the importance of a multi‐professional diagnostic work‐up and management of swallowing difficulty in an otherwise healthy teenager over the time‐course of 1.5 years (Figure 1).

## 2 CASE PRESENTATION

A 16‐year‐old girl presented to Emergency Room (ER) of Gävle Regional Hospital, Sweden, in January 2020. Her complaints were increasing swallowing problem, particularly with solid food, and globus pharyngicus (the persistent but painless sensation of a lump in the throat) for 4 days. She denied ingestion of a caustic agent or foreign bodies prior to onset of symptoms. Clinical examination by an Ear Nose Throat (ENT) specialist did not reveal anything that could explain the symptoms. The patient had no trouble swallowing saliva and she could swallow some water during the examination. Following the hospital's guidelines for acute onset dysphagia, a radiologic barium swallow test was performed the next day and was found unremarkable.

### 2.1 Evaluation

Two weeks later, the patient was seen for a follow up by the ENT. Her swallowing problems persisted. She was eating only soft foods like pudding and drinking liquids. She reported no weight loss or gastroesophageal reflux. She could now describe three choking incidences during the month prior to the first hospital visit and expressed anxiety for solid food getting stuck in her throat and falling into her trachea. The patient denied having experienced any other psychological or physical trauma. During the previous year due to self‐harm, she had had contact with the Child and Adolescent Psychiatry unit, where she was diagnosed with mild anxiety and depression. She was recommended to contact the youth guidance center, but chose not to. The clinical examination by ENT was again unremarkable. She refused fiberoptic endoscopy but agreed to assessment by a speech and language pathologist (SLP).

### 2.2 Speech and language pathologist evaluation

In February 2020, 3 weeks after the previous ENT visit, a clinical evaluation of feeding and swallowing was performed. This included evaluation of oral sensorimotor function, a questionnaire on self‐reported swallowing problems (EAT‐10)^5^ and a swallowing capacity test with a 150 ml water.^6^ The patient described that her swallowing problems had improved and that she could now eat solids, but that it took a long time. Her self‐reported weight was 54 kg and height 166 cm (Body Mass Index [BMI] 19.6). Description of the meals she had ingested the previous day showed no restrictions, managing level 7 out of 7 in the International Dysphagia Diet Standardization—framework.^7^ The oral sensorimotor examination was unremarkable but on the EAT‑10 she scored 10 out of 40 (normal <3) and she had a decreased swallowing capacity for a 150 ml water in 26.9 s (normal <15 s).

Psychosocial factors were considered. The patient was very timid. Her parents showed concern about the small amounts of food she ingested, but the patient herself did not. In addition, she described long days in school, long journey by bus to and from school and stress at lunchtime.

A video fluoroscopic swallow study (VFSS) and referral to the pediatric clinic were proposed. The patient was recommended to eat what she could and take liquid dietary supplements while the medical investigation continued. The result of the VFSS 4 weeks after the initial SLP evaluation was inconclusive since the patient only could swallow minute pieces of solid or semisolid food. Liquids were taken in small sips, but results were within normal limits. Two days after the VFSS the patient's mother called the SLP and informed her that they were very frustrated and worried since the patient refused to eat anything except the liquid dietary supplements and other liquids.

### 2.3 Evaluation from the pediatric clinic

Consultation with a general pediatrician was conducted in a team‐based meeting together with patient, her mother, SLP and a dietician. At this point the patient was getting her nutrition primarily from liquid dietary supplements. Neurological examination and evaluation of heart, throat and abdomen were normal. Laboratory work‐out and electrocardiography following the visit were unremarkable (Table 1). The patient was referred to a clinical psychologist and for a gastro‐esophagoscopy with biopsy.

**TABLE 1:** Laboratory test results April 2020

Test	Result	Reference	Unit
Biochemistry			
p‐Sodium	141	137–145	mmol/L
p‐Potassium	3.7	3.5–4.4	mmol/L
p‐Calcium	2.48	2.15–2.50	mmol/L
p‐Phosphate	1.1	1.0–1.5	mmol/L
p‐Albumin	48	36–48	g/L
p‐Creatinine	66	45–90	μmol/L
p‐Urea	3.5	2.6–6.4	mmol/L
p‐Urate	300	155–350	μmol/L
Liver			
p‐ALP	1.3	0.7–1.9	μkat/L
p‐GT	0.14	0.15–0.75	μkat/L
p‐ALAT	0.18	0.00–0.75	μkat/L
p‐LD	2.1	1.8–3.4	μkat/L
Blood count			
b‐Leucocytes	4.5	3.5–8.8	×10^3/μl
b‐Hemoglobin	129	117–153	g/L
b‐Thrombocytes	269	165–387	×10^3/μl
b‐Microscopy	Normal		
Hormonal analyses			
p‐TSH	0.6	0.4–3.7	mIE/L
p‐fT4	19.8	12–22	pmol/L
p‐fT3	6	3.9–7.7	pmol/L
Vitamins and minerals			
s‐Vitamin D	72	51–150	nmol/L
p‐Ferritin	48	10–150	noml/L
p‐Kobalamine	784	150–500	pmol/L
Allergies			
s‐Phadiatop	negative		
a‐Transglutaminase IgA	0.2	0.0–7.0	kU/L
p‐IgA total	1	0.70–3.65	g/L
Electrocadiography	Normal		

The in‐house clinical psychologist evaluated the patient's mental health. Patient appeared to be functioning well in all different areas of life, apart from eating. She performed well at school, had friends and hobbies, had no problems sleeping, and was mostly in a good mood. There was a history of self‐harming, related to conflicts with friends, but this behavior had stopped 2 years prior as noted by the ENT. As relayed to the ENT, the patient described having had anxiety symptoms while trying to swallow. On one occasion, these symptoms met the criteria for a panic attack.

### 2.4 Differential diagnosis

Differential diagnostics of dysphagia in older children and adolescents are presented in Table 2.^4^, ^8^, ^9^ Organic conditions should be separated from behavioral problems such as functional dysphagia and eating disorders.^8^ Organic conditions can further be divided into structural abnormalities of ENT region, neuromuscular conditions, cardiopulmonary and gastrointestinal tract problems, infections, metabolic diseases and inflammatory conditions.^4^, ^8^, ^9^

**TABLE 2:** Differential diagnoses of dysphagia in adolescents (adapted from ref. 4,8 and 9).

STRUCTURAL ABNORMALITIES		OTHER ORGANIC CAUSES			
*Nose and nasopharynx*	*Hypopharynx and larynx*	NEUROMUSCULAR	CARDIOPULMONARY	IATROGENIC	BEHAVIORAL FEEDING DIFFICULTIES
**Adenoid hypertrophy**	*Laryngotracheoesophageal cleft*	**Cerebral palsy**	*Congenital cardiac disease*	Need for positive pressure ventilation	Functional dysphagia**Anorexia nervosa**
Congenital intranasal masses	Glottic stenosis	*Central nervous system tumors*	Bronchopulmonary dysplasia	Prolonged intubation	**Bulimia nervosa**
Inferior turbinate hypertrophy	Laryngeal masses	*Hydrocephalus*	Thoracic surgical procedures	Prolonged parenteral or enteral tube feeds	**ARFID**
Midface hypoplasia	Subglottic stenosis	*Traumatic brain injury*		Tracheotomy	PANDAS
Nasopharyngeal masses	Vascular malformations	Arnold‐Chiari malformation			Selective eating
	Vocal fold immobility	Congenital viral infections	**GASTROINTESTINAL**	**METABOLIC**	**INFECTIONS**
***ORAL CAVITY AND OROPHARYNX***	***Trachea and esophagus***	Intraventricular hemorrhage	**Constipation**	*Urea cycle disorders*	*Botulism*
**Micrognathia or retrognathia**	Cricopharyngeal achalasia	Microcephaly	**Coeliac disease**	*Organic acidosis*	*Chagas disease*
*Cleft lip or palate (post op)*	Tracheoesophageal fistula	Multiple sclerosis	**Eosinophilic esophagitis**	*Hyperthyroidism*	*Diphteria*
Oral ties	Esophageal atresia (post op)	Muscular dystrophies	**Food allergies**		*Lyme disease*
Macroglossia	Tracheal stenosis	Myasthenia gravis	**Gastroesophageal reflux disease**	**AUTOIMMUNE/INFLAMMATORY**	*Meningitis*
High arched palate	Tracheobronchomalacia	Periventricular leukomalacia	*Ingestion of caustic agents*	*Amyloidosis*	*Neurosyphilis*
Congenital oral masses	Vascular rings and slings	Seizure disorders	*Foreign body in GI tract*	*Sarcoidosis*	*Viral infections* (e.g. *EBV*, *CMV*, *Polio*, *Herpes*)
Tongue base masses			Gastroparesis	*Systemic lupus erythematosus*	

*Note*: **Common differential diagnoses**, *Rare but important differential diagnoses that should be ruled out*.

Abbreviation: ARFID, Avoidant restrictive food intake disorder; CMV, Cytomegalovirus; EBV, Ebstain‐Barr virus; GI, Gastrointestinal; PANDAS, Pediatric autoimmune neuropsychiatric disorders associated with streptococcal infections.

Diagnosis of dysphagia is further evaluated by estimating the stage of swallowing difficulty. In our case, clinical evaluation by the SLP and VFSS revealed that the patient had most issues in the oral stage with initiating a swallow reflex. This was later confirmed with a second VFSS. Esophageal biopsies were normal so eosinophilic esophagitis and was ruled out. Physical examination and laboratory work‐up spoke against an organic cause and she showed no danger signs of organic disorder like coughing, choking, pain, recurrent pneumonia, vomiting or diarrhea.^8^

It was concluded that the patient did not meet criteria for any psychiatric diagnosis, such as depression or any kind of anxiety diagnosis according to the Diagnostic and Statistical Manual of Mental Disorders, Fifth Edition (DSM‐5).^10^ Obsessive–compulsive disorder was ruled out as a cause, since there were no obsessive thoughts or compulsive behaviors present. Neither did she meet criteria for any classical eating disorder such as anorexia nor bulimia, since she denied being afraid of weight gain and displayed no wish to be smaller. Avoidant/Restrictive Food Intake Disorder (ARFID) was considered, but was rejected due to criterion D, which stipulates that the disorder should not be explained by a simultaneous medical condition.^11^

Her medical history included sudden cessation of feeding after a traumatic event, and she described symptoms commonly associated with functional swallowing difficulties such as globus feeling and fear of choking. Specific phobia was considered as a differential diagnosis, but it was concluded the functional swallowing diagnosis better explained the symptoms. However, since she was only able to swallow small amounts during the first VFSS, an organic etiology related to hypopharynx and larynx could be ruled out only after a second VFSS was completed at the end of the treatment period.

### 2.5 Treatment

In April 2020, 4 months after the first visit to the hospital's ER, there was a 4 kg weight loss and the patient's diet consisted of liquid dietary supplements, soups processed in a blender and thin liquids. Compared to the initial SLP evaluation, her symptoms had worsened with EAT‐10 score increasing from 10 to 24 and she could only drink 50 of the 150 ml of water in the swallowing capacity test due to heart palpitations and discomfort.

An intense behavioral treatment program was commenced. There is currently no evidenced‐based treatment for functional dysphagia,^12^ but on the basis of previous cases with younger children, the Sequential Oral Sensory (SOS) feeding approach was initiated.^13^ After a pre‐treatment session, where the action of eating and swallowing was explained anatomically and physiologically with a model, it was decided that the patient should come to the SLP clinic twice per week for 4 weeks. Some sessions were arranged jointly with the psychologist. The patient should also practice every day between sessions and fill out a practice diary.

Sequential Oral Sensory therapy includes working with six aspects of eating: sight, interaction, smell, touch, taste, chewing and swallowing with a gradual exposure to feared foods. Additional techniques from cognitive behavioral therapy (CBT) were also added. These were administered by the psychologist, and included psychoeducation (learning about emotions, thoughts, sensations of the body, and behaviors), as well as behavioral and cognitive techniques. We applied Clark's cognitive model of panic^14^ to this part of the treatment as the patient displayed a fear of internal sensations similar to that seen in panic disorder. Also included were a breathing technique^15^ and interoceptive exposure, for example hyperventilating in a controlled setting.

A pulse oximeter was used to monitor heart rate for anxiety. The patient set her own treatment goals. During clinic sessions the patient was presented with increasing bolus volumes while wearing a pulse oximeter. When the pulse rate escalated, the eating was paused and the patient was asked to describe her discomfort and use box breathing.^15^ When the heart rate returned to normal the eating/swallowing recommenced.

After completing the intense treatment program in July 2020, the patient had reached all her treatment goals: she could manage all food consistencies and finish a meal within 20–30 min. However, most solid food demanded extra effort. She still needed liquid supplements to maintain her weight and she felt that eating still took too long. A treatment break during summer vacation was decided, but the patient continued to practice daily on her own as in‐home maintenance. A joint follow‐up visit with the patient and her parents was scheduled for 6 weeks later.

### 2.6 Follow‐up

When the patient returned to the clinic in August 2020 she had practiced daily. She had eaten solids such as chicken and had maintained a stable weight but had not been able to gain back the 4 kilos she had lost. Her parents were concerned about her restricted dietary intake. Two new goals were suggested: to increase the number of meals per day by one and to increase the portion size at each meal by adding one spoonful with the long‐term goal of gaining back four kilos. The patient was to continue in‐home treatment and the SLP provided a training schedule for this.

At follow‐up in September 2020 both new treatment goals had been reached and the dietary schedule was filled out. Two new goals were set: being able to eat mixed food consistencies in the same bite and decreasing the intake of liquid dietary supplements to one per day. The patient also got a new training schedule.

Due to Covid‐19 and stress with school obligations, the next follow up did not take place until 6 months later in March 2021. The patient said she had continued to push herself to eat more challenging food consistencies such as steak and she had partly managed to decrease the intake of liquid dietary supplements. However, she had lost two additional kilograms and wanted to continue the treatment. She thought out two further treatment goals: to decrease the time it takes to finish a meal and not having to chew every bite for so long.

### 2.7 Outcome

After completing the second treatment program in May2021 the patient managed to eat a meal consisting of various food viscosities in 15–20 min, which is within normal limits, and acceptable to the patient. She could swallow larger volumes (10–15 ml) and said she felt confident using the breathing techniques if she felt anxious during a meal. However, on the EAT‐10 she still scored 16 points, which was clearly above the cut‐off of three, though a clear improvement compared to 24 at the treatment start. The decision to end treatment was made because the patient, in addition to having met most of her goals, showed herself capable of continuing training on her own. No adverse or unanticipated events were observed during evaluation and treatment.

## 3 DISCUSSION

This is a case report discussing the diagnostic procedure and treatment of an adolescent girl with a sudden debut of dysphagia. Arriving at the definitive diagnosis took almost 1.5 years and required co‐operation between ENT, SLP, pediatrician, psychologist and a dietician. Our patient was diagnosed with functional dysphagia, which is a diagnosis of exclusion and relatively uncommon form of pediatric feeding disorder in adolescents.^16^, ^17^ Concurrent to diagnostic work‐up, a treatment program was commenced.

The initial diagnostic test for dysphagia has traditionally been a barium swallow in order to visualize the esophagus via contrast radiography to determine if there is a stricture, intraluminal mass, extraluminal compression or aspiration. If the results from the barium swallow test are incomplete or a more functional approach is needed, a VFSS is performed. An algorithm for evaluation of pediatric dysphagia published in 2020 suggested going straight for the VFSS or flexible endoscopic evaluation of swallowing.^4^ Further diagnostic work‐up and management depends on the stage of dysphagia identified and presence of red flags.^4^, ^8^ Our patient underwent gastro‐esophagoscopy with unremarkable findings and in the absence of alarm symptoms, magnetic resonance imaging and computer tomography of head, neck and chest were not pursued.

Evidence on management of non‐organic swallowing difficulties in older children is scarce. A systematic review of articles published before the end 2015 identified 61 studies reporting intervention strategies for pediatric dysphagia covering oral motor, sensory, pharmaceutical and behavioral domains.^12^ Level of evidence was low with only a few large randomized controlled trials published and none of these including adolescents.^12^ Another review from 2020 concentrating only on management of ARFID found equally little evidence to support any particular treatment modality.^18^

In the absence of an evidence‐based treatment, we applied the SOS therapy,^13^ with additional exercise principles that are used in motor swallowing rehabilitation: intensity, specificity and transference.^19^ Through the SOS approach we addressed sensory processing so that our patient could work on problematic factors in a step‐by‐step approach until she became accustomed to various food properties. Although the SOS approach is focused on children from 6 months to 6 years, we find that the program can be used successfully in adolescents with some minor adaptations. As the level of anxiety that the older child experiences when exposed to a difficult food consistency might not be as easy to notice as it is in young children, we used a pulse oximeter to measure the heart rate is an effective adjunct. Components from CBT were added since this method as of now is the most recommended for most anxiety‐related disorders and has also been suggested as a treatment for ARFID.^20^

Our patient had two treatment goals that she only reached partially, and they were set by the SLP and her parents: to decrease the use of liquid dietary supplements and restore her weight. Kleim and Jones have outlined 10 principles of neuroplasticity that can be helpful in a treatment environment and salience, or meaningfulness, is one of them.^21^ This underlines the fact that setting your own goals is a vital part of the intervention.

We emphasized the importance of consistently practicing at home, as is vital in any treatment focusing on lasting behavior change. Research on exposure therapy in CBT for anxiety disorders has shown that a learned fear response cannot be eradicated, but that a new, different response can be subsequently learned.^22^ For optimal learning, practice should be done as often as possible and in different settings. Our patient therefore was assigned regular homework. Her adherence to these was high, and she seemed motivated to practice at home. However, she had some difficulties formulating by herself what the next step might be. During the break before the last treatment phase, she seemed less motivated to focus on these changes, since there were other things taking priority in her life at that point.

The fact that the self‐reported symptom score, in this case the EAT‐10, was still well above the cut‐off point when treatment ended is a fairly common occurrence. Increase in patient knowledge regarding function, increased awareness and obtaining a vocabulary for a function might explain this.

In the case report presented, there were clinical guidelines to follow to some extent, but no structure for multi‐professional teamwork existed at our hospital. Such approach will be of value also for other complex functional disorders.

In conclusion, we suggest that management of swallowing disorders in adolescents should be team based. Diagnostic work‐up and evaluation of underlying etiology can be a long process whereby initiating contact with SLP, dietician, and other team members concurrently to alleviate symptoms and avoid an escalation of stress and anxiety both with parents and the adolescent patient is recommended.

## AUTHOR CONTRIBUTIONS

Author 1: Conceptualized and designed study, collected the data, wrote original draft, reviewed and edited the paper. Author 2: Collected the data, wrote the original draft, reviewed and edited the paper. Author 3: Collected the data, wrote the original draft, reviewed and edited the paper. All authors approve of the final version of the paper.

## CONSENT

Written informed consent was obtained from the patient to publish this report in accordance with the journal's patient consent policy.

## Data Availability

Data sharing not applicable ‐ no new data generated
